# The relationship between dietary inflammatory index and metabolic syndrome and its components: a case study in Kashi urban, Xinjiang

**DOI:** 10.3389/fnut.2024.1334506

**Published:** 2024-02-29

**Authors:** Yangyi Zhang, Xiangtao Liu, Yinxia Su, Yan Jiang, Junxiu Cai, Xiaoping Yang, Yuan Zou, Jing Chen, Xingyang Zhao, Hui Xiao

**Affiliations:** ^1^The First Affiliated Hospital of Xinjiang Medical University, Urumqi, China; ^2^Department of Public Health, Xinjiang Medical University, Urumqi, China; ^3^Department of Medical Engineering and Technology, Xinjiang Medical University, Urumqi, China; ^4^South University of Science and Technology Hospital, Shenzhen, China; ^5^Primary School Affiliated to Xinjiang Medical University, Urumqi, China

**Keywords:** dietary inflammatory index (DII), metabolic syndrome, Xinjiang (China), Kashi urban, nation of Uygur, diet quality

## Abstract

**Introduction:**

This paper examines the association between the dietary inflammatory index (DII) and the risk of metabolic syndrome (MS) and its components among Uygur adults in Kashi, Xinjiang.

**Methods:**

The study used the multi-stage random cluster sampling method to investigate the adult residents of Uighu aged over 18 years old in one county and one township/street of three cities in Kashi between May and June 2021. All dietary data collected were analyzed for energy and nutrient intake with a nutritional analysis software, followed by a calculation of DII. Logistic regression was used to estimate the association between DII and the risks of MS and its components.

**Results:**

The maximum DII value across our 1,193 respondents was 4.570 to 4.058, with an average value of 0.256. When we analyzed the DII as a continuous variable, we determined the anti-inflammatory diet has been identified as a mitigating factor for metabolic syndrome (OR = 0.586, 95% CI = 0.395–0.870), obesity (OR = 0.594, 95% CI = 0.395–0.870), elevated fasting glucose levels (OR = 0.422, 95% CI = 0.267–0.668), and hypertension (OR = 0.698, 95% CI = 0.488–0.996). When the model was adjusted by sex, age, and occupation, we found a significant correlation between high- and low-density lipoproteinemia and DII (OR = 1.55, 95% CI = 1.040–2.323). The present study identified four distinct dietary patterns among the population under investigation. There was a linear trend in the incidence of MS and hypertension across low, middle, and high levels of fruits and milk dietary pattern model (*p* = 0.027; *p* = 0.033), within this dietary pattern may serve as protective factors against MS and hypertension, suggesting that fruits and milk within this dietary pattern may serve as protective factors against MS and hypertension. And the linear trend in the incidence of elevated fasting glucose and obesity across the low, medium, and high scores of meet and eggs dietary pattern (*p* = 0.006; *p* < 0.001), suggest that a diet rich in meat may potentially contribute to an increased risk of developing elevated fasting glucose levels and obesity. An observed linear trend in the incidence rate of high fasting blood glucose across low, moderate, and high scores of dried fruits and nuts dietary pattern (*p* = 0.014), indicating that increased consumption of nuts acted as a protective factor against elevated fasting blood glucose levels and contributed to their reduction.

**Discussion:**

The dietary inflammation index was integrated with the findings from the study on the dietary patterns of the sampled population, revealing that an anti-inflammatory diet demonstrated a protective effect against metabolic syndrome, obesity, high fasting blood glucose, and hypertension in this specific population. laying the foundation for further research.

## 1 Introduction

Metabolic syndrome (MS) is a cluster of conditions stemming from central obesity, including hypertension, dyslipidemia, impaired glucose tolerance, diabetes, and other metabolic abnormalities. MS involves a group of risk factors that are particularly important for the development of cardiovascular disease. Its prevalence in China has risen together with economic growth and subsequent changes in lifestyle. In 1992, the prevalence rate of MS was estimated at 13.3% by a cohort study covering 27,739 adults in 11 provinces and cities (1). The results of the cross-sectional survey of Chinese adults conducted by the Asian International Cardiovascular Disease Cooperation Group in 2000–2001 showed that the prevalence rate of MS in China had risen to 16.5% (2), and by 2013, Wang et al. (3) estimated it at 33.9% among urban residents.

Many epidemiological and clinical studies show that chronic and low-grade systemic inflammatory reaction may be the core point of MS pathogenesis and the connecting link between mutual transformation and interaction of various components, and is part of the initiation factor of MS-insulin resistance or hyperinsulinemia (4). At present, an increasing amount of evidence indicates that different dietary patterns, foods, and nutrients have anti-inflammatory or pro-inflammatory effects, suggesting that optimizing dietary structure can help improve chronic low-grade inflammation.

Therefore, some studies have used the population-based dietary inflammatory index (DII) to obtain the potential inflammatory factors in individual diets (5). The School of Public Health of the University of South Carolina summarizes all the literature and data relevant to the effect of common dietary ingredients/nutrients on serum inflammatory markers from 1950 to 2010, and calculates the inflammatory effect index of each dietary ingredient/nutrient (6, 7). The paper uses official data and relevant literature referring to 11 countries, to calculate (i) the average daily intake and standard deviation of the global average daily intake of common dietary ingredients/nutrients of the population, and (ii) their DII according to the daily dietary intake of the respondents, and provide an effective tool for accurate and quantitative evaluation of the level of dietary anti-inflammation/pro-inflammatory (6, 7). At present, many scholars use DII to evaluate cardiovascular disease, metabolic disease, cancer, and COPD (8–11), and think that the DII score can provide accurate insight into the potential of dietary inflammation and better explain the relationship between diet, inflammation, and cardiovascular metabolic disease.

Many population studies suggest that the dietary structure of the Uyghur population is questionable: with its high intake of calories, protein and fat and low intake of vitamins and trace elements, it is little wonder that the prevalence of MS has been estimated at 30–35% (12). Hitherto, no study has examined the impact of the overall anti-inflammatory/pro-inflammatory tendency on the MS of ethnic minorities in China. This study uses the DII developed by the School of Public Health of the University of South Carolina to investigate the overall dietary inflammation of Xinjiang Uygur residents and its contributing factors. The aim is to evaluate the dietary anti-inflammatory/pro-inflammatory tendency of MS and explore the relationship between DII and MS and its components in order to propose effective evaluation indicators for clinical prevention and treatment of MS, and provide a solid scientific reference for government departments to improve nutritional policy, dietary guidance, nutritional intervention, and research the pathogenic factors related to chronic diseases.

## 2 Object and method

### 2.1 Research object

The study was backed by empirical evidence and substantial financial support from the Xinjiang Uygur Autonomous Region Natural Science Foundation (the correlation between dietary patterns, TCF7L2 gene interaction, and diabetes mellitus in Xinjiang Uygur population, Project Number: 2016D01C242) and the Youth Research Voyage Project of the First Affiliated Hospital of Xinjiang Medical University (Study on the correlation between dietary inflammatory index and inflammatory factors of metabolic syndrome and glycolipid metabolism in Urumqi population, 2022YFY-QKQN-27). The subjects used the multi-stage stratified cluster random sampling method. Between May and June 2021, Kashgar City and Shule County were selected from the Kashgar region (one city and 11 counties) for this study, and then two townships (Haohan Township, Kashgar City, Tazihong Township, Shule County) and one street office (Chasa Street Office, Kashgar City) were randomly pickedfrom 28 townships/towns and streets, and three administrative villages and communities were selected from each township/town or street. A total of 1,193 Uighur adult residents≥18 years old in the survey site were investigated. We excluded pregnant women, nursing mothers, individuals on anti-stress medication, and others with purposefully differentiated dietary habits (such as fasting during Ramadan or attending weddings and services).

### 2.2 Data collection and methods

#### 2.2.1 Data collection

(1) A questionnaire and dietary survey. The former collected basic information, such as sex, age, marital status, education level, occupation, and other demographic characteristics. The dietary questionnaire was mainly based on the *24-h dietary review questionnaire* used in the 2002 survey of the dietary habits and health of Chinese residents (6), revised to consider the local characteristics of Xinjiang and the Uighur diet in Kashgar. We used a quasi-quantitative food frequency questionnaire (SQFFQ), modified to reflect the characteristics of the Uighur diet. The questionnaire took its final form after reliability and validity tests (13). During face-to-face interviews, the respondents were asked about the frequency and consumption of various foods in the past 12 months. Data sorting led to the creation of 12 food groups: grain, vegetables, fruits, beans and their products, milk and its products, meat, eggs, nuts and dried fruits, beverages, salt oil and other foods (excluding health food).
(2) Physical measurement and blood sample collection. Physical measurement was based on the 2002 survey standard of nutrition and health status of Chinese residents (6), and height (cm), weight (kg), waist circumference (WC, cm), hip circumference (HC, cm), blood pressure (SBP, mmHg), diastolic pressure (DBP, mmHg), and other indicators were collected; the body mass index (BMI = height (kg)/weight (m)^2^) and waist to hip ratio (WHR = waist circumference (cm)/hip circumference (cm)) were calculated according to the corresponding formula. After collecting 5 mL of venous blood from the elbow of the subjects and centrifuging, the relevant biochemical indexes of the serum were determined, including fasting blood glucose (FPG, mmol/L), total cholesterol (TC, mmol/L), triglycerides (TG, mmol/L), high-density lipoprotein cholesterol (HDL-C, mmol/L), and low-density lipoprotein cholesterol (LDL-C, mmol/L).

#### 2.2.2 Survey method

(1) A face-to-face questionnaire survey. The investigator explained the purpose of the survey to the respondents, obtained their consent in signed consent forms, then read through the questionnaire to respondents and filled in the responses. For the dietary survey, we used the continuous 3d-24 h retrospective inquiry method to collect the type and intake of all foods and cooking methods for each respondent in the previous three days to estimate the intake of edible salt and oils from the foods listed by each individual. In combination with the food weighing method, a random household survey was carried out in the families of some individuals in our sample to determine their families’ consumption of edible salt and oil in the past month, which was distributed evenly to individuals according to the number of family population. Then we calculate the average daily food intake of each group.
(2) Physical examination in the township/town or street health centers where the survey took place. The investigators and medical staff who had received training in the physical examination standards conducted standardized measurements of the respondents’ physical traits (as listed in 1.2.1 above). Height and weight were measured by a domestic height scale that was calibrated before use; respondents were asked to take off their hats, shoes, and clothes and assume a standing position of 30–40 degrees with their feet evenly distributed. Our team noted the average value after two consecutive measurements (the height measurement is accurate to 0.1 cm and the weight measurement to 1 kg) and calculated BMIs accordingly. The waist and hip circumference were measured with an inelastic soft leather ruler (with a minimum scale of 1 mm). Before taking measurements, we asked the respondents to wear thin underwear, and fully expose their abdomen and buttocks. Waist circumference was measured close to but not pressuring the skin through the middle point of the line between the lower edge of the 12th rib of the anterior superior iliac spine and the iliac crest in the midaxillary line. Hip circumference was measured at the maximum circumference of the hip (i.e., the most convex part of the pubic symphysis and the gluteus maximus). Our team noted the average value after two consecutive measurements (readings are accurate to 0.1 cm). We measured blood pressure with a domestic desktop sleeve mercury sphygmomanometer. After the subjects sat comfortably in a quiet room for 5 min, they were asked to assume a sitting position to measure the blood pressure of the right brachial artery. The first and fifth tones of Koch’s were SBP and DBP, respectively. Their blood pressure was measured thrice (accurate to 1 mmHg; 1 mmHg = 0.133 kPa) with one-minute intervals between measurements. If the difference between any two measurements was more than 10 mmHg, we took a fourth measurement and used the average value of all four measurements.
(3) We collected 5 mL blood samples of elbow vein blood from all respondents, whom we asked to fast for more than 8 h, and centrifuged them at 4,500 r/min within 2 h after collection. All separated serum samples were measured in a Hitachi 7,600 Automatic Analyzer at the Laboratory of Kashgar People’s Hospital for relevant biochemical indicators (FPG, TC, TG, HDL-C, LDL-C5). The equipment was operated by the same group of professional inspectors above the technician in charge, and the kit was provided by the Northern Institute of Biology. FPG was determined with the use of the GOP-POD method, serum TC and TG by terminal colorimetry, and serum HDL-C and LDL-C by selective melting.

#### 2.2.3 Relevant definitions of MS

MS Research Collaboration Group of Diabetes Society of Chinese Medical Association, Recommendations on MS of Diabetes Society of Chinese Medical Association in 2004 (7), The individuals who satisfy all three criteria and possess all components are deemed to have metabolic syndrome: (1) overweight and/or obesity: BMI ≥ 25 kg/m^2^; (2) Abnormal blood lipids: TG ≥ 1.7 mmol/L and/or HDL-C < 0.9 mmol/L (male) or < 1.0 mmol/L (female); (3) Hypertension: systolic/diastolic blood pressure ≥ 140/90 mmHg and/or confirmed as hypertension and treated; (4) Hyperglycemia: FPG ≥ 6.1 mmol/L and (or) 2 h postprandial blood glucose (2hPG)>7.8 mmol/L and (or) those who have been confirmed as type 2 diabetes and have been receiving treatment.

#### 2.2.4 Calculation of the dietary anti-inflammatory index

The following DII formula developed by the University of South Carolina was used to calculate the DII of the respondents (6): DII of a certain dietary component/nutrient = (daily intake of this dietary component or nutrient—average daily intake of this dietary component or nutrient *per capita* in the world)/standard deviation of daily intake of this dietary component or nutrient *per capita* in the world × Inflammatory effect index of this dietary component or nutrient. Then, we summarize the DII of various dietary components/nutrients in the diet, which is the total score of DII. The higher the positive value of DII, the stronger the tendency to promote inflammation; The higher the negative value of DII, the stronger the anti-inflammatory tendency.

The final dietary inflammation index (DII) comprised 45 dietary components or nutrients. Among these, 9 components (energy, carbohydrates, protein, fat, cholesterol, iron, vitamin B12, saturated fatty acids and trans fatty acids) were found to have pro-inflammatory properties while the remaining 36 components exhibited anti-inflammatory properties. The intake of these 45 dietary components was determined through dietary surveys. Subsequently, individual component intakes were integrated to assess the overall inflammatory potential of the diet. Due to limitations in our current version of nutrition calculator software used for this study, we calculated the daily intake of more than 20 common dietary components/nutrients in the respondents’ diet and three-day data with the use of dietary nutrition analysis software, and measured for energy, protein, fat, carbohydrate, dietary fiber, iron, zinc, selenium, magnesium, VitA, VitC, VitE, VitB1, VitB2, niacin, cholesterol, folic acid, saturated fatty acid, monounsaturated fatty acid, and polyunsaturated fatty acid. We used the average daily intake of dietary ingredients/nutrients during the three-day period to calculate the DII value of the respondents using the DII formula. Then, according to the total quartile of DII, P25, P50 and P75 were divided into four groups. Q1 was the anti-inflammatory tendency group (DII < −2.029), Q2 (DII 2.029 ~ 0.374), Q3 (DII 0.374 ~ 2.514), and Q4 were the pro-inflammatory tendency group (DII>2.514).

### 2.3 Quality control

(1) Given the unique dietary habits of the Uygur residents in Kashgar, a preliminary survey and demonstration were conducted to tailor the content of our dietary survey to the actual diet of the respondents. Considering that the daily dietary structure of the individuals in our sample varies during the week, we adjusted the period to reflect both workdays and weekends. We collected the respondents’ daily tableware and food as food models, using different food models to help them judge their food types and intake.
(2) We ensured that all personnel who conducted the physical examinations were relatively fixed, and all instruments and equipment were used only after appropriate calibration. The collection of blood samples was conducted by personnel assigned in strict accordance with quality control standards. All relevant operations were carried out by personnel assigned in strict accordance with the instructions and requirements of the experiment. All blood samples were subject to quality control in batches.

Data input was delegated to two persons, and the logical error correction function was set in the database to prompt in real time and correct in time. The obvious illogical outliers were eliminated, missing values within the allowable range were statistically processed, and possible confounding factors, such as age and sex, were controlled through hierarchical analysis.

### 2.4 Data processing method

Epidata3.0 software was used to input the original data, and each subject’s daily intake of each nutrient was calculated using the nutrition calculator V2.65 standard version (prepared by the China Center for Disease Control and Prevention). We used MS Excel for data collation and the SPSS17.0 statistical software for data analysis. Measurement data consistent with the normal distribution were represented by the mean; the data of non-normal distribution were represented by the median (interquartile interval), and its natural logarithm was taken for statistical analysis. With the quartile level of the DII as the independent variable and MS and its components as the dependent variable, we analyzed the relationship between DII and MS and its components by using a two-class logistic regression model; we adjusted and controlled the confounding factors such as age, sex, occupation, and others, as covariates in the model fitting. The difference was statistically significant with *p* < 0.05.

## 3 Results

### 3.1 Sample parameters

A total of 1,193 people were surveyed, aged between 18 and 91, with an average 45.18 ± 15.010 years. The sample included 485 men (40.7%) with an average age 47.66 ± 15.737 years, and 708 women (59.3%) with an average age 43.48 ± 14.254 years. A total of 1,113 people (94.3%) were married or remarried; 56 were unmarried (4.7%), and 24 divorced or widowed (2%). 671 (56.2%) were farmers; 335 declared themselves unemployed (35.3%), 161 (13.5%) in employment and 26 (2.2%) retired. 1,027 (86.1%) had education below the high-school level, and 166 people (13.9%) had high-school level education or above. 448 respondents (37.6%) were city dwellers and 745 (62.4%) lived in rural areas (full presentation in Table 1). 429 adults with metabolic syndrome in Kashgar, Xinjiang, were investigated, with a prevalence rate of 35.96%; 158 cases of high-altitude abdominal glucose with a prevalence rate of 13.24%; 195 cases of hypertension (prevalence rate: 16.35%); 766 cases of obesity (prevalence rate: 64.42%); 527 cases of hypertriglyceridemia (prevalence rate: 44.17%); 362 people with high-density lipoprotein disease (prevalence rate: 30.34%) Full data are illustrated in Table 2.

**Table 1:** Sample characteristics (*n*, % or $\bar{x}\pm s$ ).

Category	MS	Non-MS	*p*
	(*n* = 429)	(*n* = 764)	
Gender			
Male	114 (26.6)	289 (37.8)	<0.001**
Female	315 (73.4)	475 (62.2)	
Place of residence			
Urban	206 (48.0)	319 (41.8)	0.061
Rural areas	223 (52.0)	445 (58.2)	
Degree of education			
Below high school	390 (90.9)	665 (87.0)	0.056
High school and above	39 (9.1)	99 (13.0)	
Occupation			
Farmer	203 (47.3)	400 (52.4)	<0.001**
Other working or unemployed	126 (52.7)	364 (47.6)	
Age (average)	47.84 ± 10.94	43.39 ± 13.88	–

Chi-square test was used for counting data and t-test for measuring data. **p < 0.001, with significantly different results.

**Table 2:** Physical examination and DII of the respondents (*n*, % or $\bar{x}\pm s$ ).

Category	MS	Non-MS	*p*
	(*n* = 429)	(*n* = 764)	
BMI(kg/m^2^)	30.57 ± 4.30	26.02 ± 4.89	<0.001^**^
WHR	0.95 ± 0.07	0.89 ± 0.08	<0.001^**^
TG (mmol/L)	2.48 ± 1.25	1.44 ± 0.88	<0.001^**^
HDL (mmol/L)	1.07 ± 0.22	1.14 ± 0.24	<0.001^**^
SBP (mmHg)	139.97 ± 20.51	113.56 ± 18.92	<0.001^**^
DBP (mmHg)	90.31 ± 13.60	74.11 ± 12.51	<0.001^**^
FPG (mmol/L)	7.40 ± 3.09	5.44 ± 1.72	<0.001^**^
DII	0.403 ± 3.49	(−0.386) ± 3.972	0.003^**^

BMI is the body mass index; WHR is the Waist-to-Hip Ratio; TG is the triglyceride; HDL is high density lipoprotein; SBP and DBP are the systolic and diastolic blood pressure; FPG is the fasting blood glucose; DII indicates the dietary inflammatory index. **p < 0.001; results are significantly different.

### 3.2 DII, MS, and its components

Our physical and laboratory examinations show that the DII in our sample varies from Q1-Q4 (anti-inflammatory grading to pro-inflammatory grading) to obesity (*χ*^2^ = 9.825, *p* = 0.020), high fasting blood glucose or not (*χ*^2^ = 15.390, *p* = 0.002), with or without metabolic syndrome (*χ*^2^ = 15.626, *p* = 0.001) was statistically significant. We found no statistically significant difference between the DII components with or without hypertriglyceridemia, with or without low high-density lipoprotein, and with or without hypertension (see Table 3).

**Table 3:** DII grades, MS, and its components in our sample of Uygur adults (*n*, %).

MS and its components		DII classification				Total	*χ* ^2^	*p*
		Q1 (<-2.029)	Q2 (−2.029–0.374)	Q3 (0.374–2.514)	Q4 (>2.514)			
Obesity	Y	125 (29.3)	110 (25.8)	101 (23.7)	91 (21.3)	427	9.825	0.020^*^
	N	171 (22.3)	189 (24.7)	197 (25.7)	209 (27.3)	766		
Hypertriglyceridemia	Y	178 (26.7)	164 (24.6)	159 (23.9)	165 (24.8)	666	4.569	0.206
	N	114 (21.6)	136 (25.8)	143 (27.1)	134 (25.4)	527		
Hypohigh-density lipoprotein	Y	198 (23.8)	213 (25.6)	209 (25.2)	211 (25.4)	831	3.381	0.337
	N	104 (28.7)	85 (23.5)	89 (24.6)	84 (23.2)	362		
High fasting blood glucose	Y	283 (27.3)	260 (25.1)	243 (23.5)	249 (24.1)	1,035	15.390	0.002^*^
	N	22 (13.9)	39 (24.7)	51 (32.3)	46 (29.1)	158		
hypertension	Y	261 (26.2)	254 (25.5)	244 (24.4)	239 (23.9)	998	3.115	0.374
	N	42 (21.5)	46 (23.6)	52 (26.7)	55 (28.2)	195		
MS	Y	204 (26.7)	195 (25.5)	180 (23.6)	185 (24.2)	764	15.626	0.001^**^
	N	78 (18.2)	100 (23.3)	131 (30.5)	120 (28.0)	429		

*p < 0.05, **p < 0.001, and results are significantly different. In this chart, “Y” represents the presence of metabolic syndrome (MS), while “N” indicates its absence. Revised sentence: Please refer to Section 2.2.3 for the diagnostic criteria of MS.

### 3.3 OR value of DII and risk of MS and its components

Our research shows that the maximum DII value across our 1,193 respondents was 4.570 to 4.058, with an average value of 0.256. When we analyzed the DII as a continuous variable, we revealed the anti-inflammatory diet has been identified as a mitigating factor for metabolic syndrome (OR = 0.586, 95% CI = 0.395–0.870), obesity (OR = 0.594, 95% CI = 0.395–0.870), elevated fasting glucose levels (OR = 0.422, 95% CI = 0.267–0.668), and hypertension (OR = 0.698, 95% CI = 0.488–0.996). When the model was adjusted by sex, age, and occupation, we found a significant correlation between high-and low-density lipoproteinemia and DII (OR = 1.55, 95% CI = 1.040–2.323; as in Table 4).

**Table 4:** DII and OR values of MS and its components.

Group	Case (*n*)	OR^a^	95%CI	OR^b^	95%CI
	MS				
Q1	78	1		1	
Q2	100	***0.586***	***0.395–0.870***	0.966	0.617–1.512
Q3	131	0.787	0.541–1.144	1.018	0.688–1.507
Q4	120	1.118	0.783–1.596	1.379	0.954–1.994
	Obesity				
Q1	171	1		1	
Q2	189	**0.594**	**0.432–0.817**	1.121	0.776–1.619
Q3	197	0.749	0.543–1.033	1.049	0.746–1.474
Q4	209	0.842	0.610–1.163	1.057	0.756–1.477
	Hypertriglyceridemia				
Q1	114	1		1	
Q2	136	0.784	0.568–1.083	1.184	0.819–1.710
Q3	143	1.021	0.745–1.401	1.269	0.911–1.768
Q4	134	1.117	0.816–1.530	1.319	0.954–1.824
	Hypohigh-density lipoprotein				
Q1	104	1		1	
Q2	85	1.316	0.924–1.873	***1.554***	***1.040–2.323***
Q3	89	0.996	0.692–1.434	1.073	0.736–1.566
Q4	84	1.066	0.743–1.530	1.067	0.737–1.543
	High fasting blood glucose				
Q1	22	1		1	
Q2	39	**0.422**	**0.267–0.668**	0.695	0.415–1.164
Q3	51	0.83	0.559–1.233	1.082	0.712–1.644
Q4	46	1.154	0.792–1.681	1.47	0.992–2.178
	Hypertension				
Q1	42	1		1	
Q2	46	**0.698**	**0.488–0.996**	1.103	0.722–1.684
Q3	52	0.785	0.553–1.114	0.991	0.678–1.449
Q4	55	0.937	0.665–1.319	1.216	0.844–1.752

a Indicates the establishment of a logistic regression model with DII index.

b Signifies a logistic regression model based on gender, age, occupation, and DII index.

The statistically significant results are indicated by the bold part with p < 0.05. An odds ratio (OR) less than 1 and a 95% confidence interval (CI) ranging from 0 to 1 (excluding 1) suggest a potential protective factor for the corresponding disease, while an OR greater than 1 and a CI greater than 1 (excluding 1) indicate a possible risk factor for the corresponding disease.

### 3.4 Dietary patterns, MS and its components

A total of four factors with eigenvalues greater than 1 were extracted through factor analysis, resulting in a cumulative contribution rate of 65.93%. This suggests that the extracted common factors effectively capture the variability within each food group. The four derived models from this analysis include: a grain and vegetable-based dietary model, a fruit and milk-based dietary model, a meat and egg-based dietary model, a dried fruit and nut-based dietary model. The results of the multiple logistic regression analysis indicated no significant association between the grain-vegetable dietary pattern and MS or its components. The consumption of fruit and milk was inversely associated with the prevalence of multiple sclerosis (MS) and hypertension, with individuals having a high intake showing 0.41 times and 0.33 times higher risk for MS and hypertension compared to those with low intake (95% CI 0.22–0.87, 95% CI 0.25–0.92). Furthermore, there was a linear trend in the incidence of MS and hypertension across low, middle, and high levels of this dietary pattern model (*p* = 0.027; *p* = 0.033), suggesting that fruits and milk within this dietary pattern may serve as protective factors against MS and hypertension. The meat-egg dietary pattern did not exhibit a significant association with multiple sclerosis (MS); however, it demonstrated a positive correlation with elevated fasting glucose levels and obesity. Individuals with high consumption had 1.35 times higher odds of having elevated fasting glucose (95% CI 0.89–2.84) and 3.26 times higher odds of being obese (95% CI 2.21–5.71), compared to those with low consumption levels. Furthermore, there was a linear trend in the incidence of elevated fasting glucose and obesity across the low, medium, and high scores of this dietary pattern (*p* = 0.006; *p* < 0.001). These findings suggest that a diet rich in meat may potentially contribute to an increased risk of developing elevated fasting glucose levels and obesity. The dried fruits and nuts dietary pattern demonstrated a significant inverse association with elevated fasting blood glucose levels. Specifically, individuals consuming a higher quantity of nuts had a 0.20 times lower prevalence of high fasting blood glucose compared to those with low nut intake (95%CI 0.12–0.75). Furthermore, there was an observed linear trend in the incidence rate of high fasting blood glucose across low, moderate, and high scores of this dietary pattern (*p* = 0.014), indicating that increased consumption of nuts acted as a protective factor against elevated fasting blood glucose levels and contributed to their reduction. The specifics are presented in Table 5.

**Table 5:** The relationship between various dietary patterns and metabolic syndrome and its components.

Dietary pattern	The level of intake	MS and its components					
		MS	High fasting blood glucose	Hypertension	Obesity	Hypertriglyceridemia	High-density lipoprotein anemia
Type of grain and vegetable	Q_1_^a^	1.00	1.00	1.00	1.00	1.00	1.00
	Q_2_	1.36 (0.72–2.98)	0.73 (0.46–1.76)	1.05 (0.62–2.49)	1.08 (0.64–2.32)	1.16 (0.61–2.51)	1.01 (0.52–2.16)
	Q_3_	1.39 (0.78–3.05)	0.69 (0.53–1.25)	1.45 (0.67–2.55)	1.25 (0.71–2.63)	1.38 (0.85–2.82)	1.45 (0.91–2.98)
	P	0.306	0.237	0.408	0.649	0.239	0.216
Type of fruit and milk	Q_1_	1.00	1.00	1.00	1.00	1.00	1.00
	Q_2_	0.47 (0.25–0.91)	0.63 (0.37–1.59)	0.56 (0.31–0.98)	0.97 (0.58–1.67)	0.89 (0.47–1.65)	1.64 (0.75–2.08)
	Q_3_	0.41 (0.25–0.91)	0.52 (0.25–0.91)	0.45 (0.25–0.91)	0.83 (0.25–0.91)	0.61 (0.25–0.91)	2.28 (0.25–0.91)
	P	0.027*	0.081	0.033*	0.432	0.187	0.279
Type of meet and eggs	Q_1_	1.00	1.00	1.00	1.00	1.00	1.00
	Q_2_	1.07 (0.61–2.33)	1.23 (1.85–2.49)	1.45 (0.92–3.42)	2.54 (1.54–4.25)	1.26 (0.89–2.95)	1.14 (0.73–2.15)
	Q_3_	1.58 (0.98–3.02)	1.35 (1.89–2.84)	1.63 (0.98–3.54)	3.26 (2.21–5.71)	1.38 (0.93–3.12)	1.34 (0.95–2.73)
	P	0.256	0.006**	0.384	<0.001**	0.349	0.385
Type of dried fruits and nuts	Q_1_	1.00	1.00	1.00	1.00	1.00	1.00
	Q_2_	0.74 (0.41–1.58)	0.33 (0.19–0.81)	0.45 (0.31–1.58)	0.73 (0.49–1.67)	0.61 (0.32–1.53)	1.82 (0.91–3.25)
	Q_3_	0.65 (0.29–1.36)	0.20 (0.12–0.75)	0.36 (0.18–1.22)	0.67 (0.42–1.55)	0.49 (0.36–1.35)	2.28 (0.98–3.58)
	P	0.093	0.014*	0.071	0.474	0.432	0.487

* indicates statistical significance at *p* ≤ 0.05; ** indicates statistical significance at *p* ≤ 0.01.

a Indicates the dietary patterns were categorized into low, medium, and high intake levels, with Q1 serving as the reference group.

## 4 Discussion

The Uygurs generally like to eat red meat, preferably beef and mutton (smoked or stewed), which abound in Xinjiang, whereas their white meat and seafood intake is generally low. Our analysis of their dietary habits shows that nuts and dried fruits with high factor load values remain part of their weekly diet. Our study confirms higher values for the factor load of salt and oil food groups, which were included in the first matrix of the Uygur dietary pattern. Some studies have shown that fruits are rich in dietary fiber, minerals, antioxidant substances, and vitamins. In addition, a reasonable daily fruit intake can reduce the risk of diabetes and cardiovascular disease (14). However, in view of the sample size and other factors, the study found no difference in fruit intake between individuals with more than three items and their related components and individuals without MS and its related components abnormalities (15). Moreover, milk and dairy products are rich sources of calcium. Some studies have found that they may reduce blood pressure and obesity, while Khan et al. (16) found that the intake of milk and its products are negatively correlated with waist circumference, hypertension, and MS. Back to our study, after adjusting the calcium intake, we say the OR value dropped, but the difference remained statistically significant (*p* < 0.05). The Fifth National Health and Nutrition Survey in South Korea showed that (17) the prevalence of MS in individuals with a high intake of milk or yoghurt was significantly lower than that with low intake (*p* < 0.001).

Our study also determined a positive correlation between the dietary pattern of meat-and-eggs type and high fasting blood glucose and obesity. The high blood sugar of meat eaters is almost ubiquitous, which may be related to the fact that a high meat intake tends to reduce glucose tolerance. Cantero et al. (18) conducted a multifactorial analysis of the dietary risk factors of obesity among 871 Hispanic and 1,599 non-Hispanic white women in the United States, and found that the prevalence of obesity was increased due to the western dietary pattern, rich in saturated fatty acid and energy intake, dominated by red meat and refined grain intake. However, a different diet pattern with a higher intake of vegetables, fruits, low-fat milk, and whole grains, and a lower intake of meat and beverages, can reduce obesity. Abe et al. (19) Among the male population, a multi-food and beverage dietary pattern containing medium-energy cheese and fat meat increases the risk of hyperglycemia and central obesity, while a fiber/bread dietary pattern rich in high-energy fiber reduces the risk. For the female population, a diet rich in white bread increases the risk of hyperinsulinemia, whereas a diet rich in milk and fat reduces the risk of hyperinsulinemia (20). Luo Tao et al. (21) found that high DII values correlate with an increased risk of cardiovascular metabolic diseases in Xinjiang’s multi-ethnic population. The key to controlling the impact of DII on cardiovascular metabolic diseases is to help a population group maintain moderate levels of waist circumference, blood pressure, and LDL. This study provides a pilot reference for future inflammatory investigations on local Uygurs population incorporating the genomics and metageomics data.

The dietary inflammation index was integrated with the findings from the study on the dietary patterns of the sampled population, revealing that an anti-inflammatory diet demonstrated a protective effect against metabolic syndrome, obesity, high fasting blood glucose, and hypertension in this specific population. Analysis of dietary patterns suggested that fruits and dairy products may possess anti-inflammatory properties for individuals with metabolic syndrome and hypertension, while nuts were identified as potential anti-inflammatory foods for those with high fasting blood glucose. Conversely, meat was found to be a pro-inflammatory food source for individuals with high fasting blood glucose and obesity. In a comprehensive survey on dietary habits and metabolic syndrome in China, it was discovered that adherence to the traditional diet rich in fruits, vegetables, and aquatic products plays a crucial role in preventing and managing metabolic syndrome (22). The findings of a meta-analysis examining the association between dairy consumption and the incidence of metabolic syndrome suggest that an increased intake of dairy products may potentially mitigate the risk of developing metabolic syndrome (23). A certain finding suggested that of the 6 common types of milk consumed, semi-skimmed and soya milk products were protective against essential hypertension, whereas skim milk had the opposite effect (24). The study conducted in Urumqi, Xinjiang on middle-aged and elderly individuals revealed that the appropriate consumption of nuts exhibited a protective effect against fasting blood glucose abnormalities (25). Numerous studies have consistently demonstrated that excessive consumption of meat, particularly red meat (such as lamb, beef, and pork), is associated with an increased risk of obesity, hypertension, hyperlipidemia, and abnormal blood sugar levels (26–28).

Some limitations of this study must be acknowledged. First, to create our sample we relied on voluntary participants willing to undergo a physical examination and provide ample dietary data. Second, fieldwork took place during the spring and summer, but dietary intake may be affected by seasonal changes; moreover, our 24-h dietary regression method for three consecutive days cannot fully represent long-term dietary habits. Finally, the survey primarily targets the general population who willingly participate in community or town physical examinations, spanning from 18 to 90 years of age, with notable variations in demographic characteristics. Following age stratification, the sample size within each stratum is relatively small, potentially impacting the representativeness of the samples. Subsequent research will delve into a comprehensive analysis of the multi-ethnic population and explore factors such as ethnicity, age, and other variables during stratification.

## Data availability statement

The original contributions presented in the study are included in the article/supplementary materials, further inquiries can be directed to the corresponding author or the primary author.

## Ethics statement

The studies involving humans were approved by the First Affiliated Hospital of Xinjiang Medical University. The studies were conducted in accordance with the local legislation and institutional requirements. The participants provided their written informed consent to participate in this study.

## Author contributions

YaZ: Conceptualization, Data curation, Formal analysis, Funding acquisition, Investigation, Methodology, Project administration, Software, Supervision, Writing – original draft. XL: Conceptualization, Data curation, Formal analysis, Funding acquisition, Investigation, Methodology, Project administration, Software, Supervision, Validation, Writing – original draft. YS: Conceptualization, Data curation, Formal analysis, Investigation, Software, Writing – original draft. YJ: Conceptualization, Investigation Methodology, Project administration, Writing – original draft. JuC: Formal analysis, Methodology, Supervision, Writing – original draft. XY: Conceptualization, Formal analysis, Methodology, Writing – review & editing. YuZ: Conceptualization, Formal analysis, Supervision, Validation, Writing – original draft. JiC: Funding acquisition, Methodology, Validation, Writing – review & editing. XZ: Methodology, Project administration, Writing – original draft. HX: Conceptualization, Data curation, Formal analysis, Funding acquisition, Software, Writing – review & editing.
